# A Longitudinal Study of Post-Traumatic Growth and Psychological Distress in Colorectal Cancer Survivors

**DOI:** 10.1371/journal.pone.0139119

**Published:** 2015-09-29

**Authors:** Stefano Occhipinti, Suzanne K. Chambers, Stephen Lepore, Joanne Aitken, Jeff Dunn

**Affiliations:** 1 Menzies Health Institute of Queensland, Griffith University, Gold Coast, Australia; 2 Centre for Cancer Research, Cancer Council Queensland, Brisbane, Australia; 3 Health & Wellness Institute, Edith Cowan University, Perth, Australia; 4 Centre for Clinical Research, University of Queensland, Brisbane, Australia; 5 Department of Social and Behavioral Sciences, College of Public Health, Temple University, Philadelphia, Pennsylvania, United States of America; 6 School of Public Health, Queensland University of Technology, Brisbane, Australia; 7 School of Social Science, University of Queensland, Brisbane, Australia; University of Toledo, UNITED STATES

## Abstract

The stability of post-traumatic growth over time and the relationship between post-traumatic growth and traditional distress outcomes remains unclear. We tracked post-traumatic growth in a population-based sample of colorectal cancer patients from soon after diagnosis to five years subsequently to assess the heterogeneity of a post-traumatic growth response to cancer over time and describe the simultaneous and longitudinal relationships between post-traumatic growth and psychological distress. 1966 colorectal patients who were five months post diagnosis were assessed six times over a five year period. There was considerable heterogeneity associated with both psychological distress and benefit finding scores over time. However, both for benefit finding and psychological distress, the variation in individual scores suggested an underlying positive linear trend and both lagged and lagged change components. Specifically, benefit finding and psychological distress are mutual leading indicators of each other. First, benefit finding served as a leading indicator of distress, in that increases in reported benefit finding from year to year predicted higher future increases in psychological distress. As well, in an inverse relationship, psychological distress served as a leading indicator of benefit finding, such that increases in reported distress from year to year predicted lower future increases in benefit finding. Post-traumatic growth may reflect patients coping efforts to enhance perceptions of wellbeing in response to escalating cancer–related threats, acting as harbinger of increasing trajectories of psychological distress. This explanation is consistent with a cognitive dissonance response in which threats to the integrity of the self then lead to a tendency to accentuate positive aspects of the self.

## Introduction

Post-traumatic growth (PTG) has become the focus of intensive research efforts in recent years. Although there is some controversy over precisely what constitutes PTG [1, 2], definitions tend to converge around a process or mechanism by which people who have suffered highly adverse, traumatic events derive a paradoxical, multifaceted benefit from such exposure. Such benefits may include: improved psychological adjustment; closer connections with family and friends; and self-perceived spiritual enhancement. PTG researchers have advanced a perspective that challenging, threatening and even traumatic life events may cue the desire to better the self in some way [3, 4]. That is, people may come to accept that an objectively aversive event, such as the diagnosis of a life threatening illness or the experience of an acute catastrophe in war, may well provide a chance to reevaluate life priorities and other facets of adjustment, usually for the better (i.e., benefit finding; BF). Further, the disruptive effect of a threating event is seen to set in train an ongoing re-evaluation of and reengagement with the events and people in the person’s life (i.e., sense making). It may then be that any effects of post-traumatic growth will not be apparent until some time after the initial triggering event.

Over the past two decades, the nature, stability and predictive power of the construct of post-traumatic growth have been the center of a lively academic debate (for a review with commentary that examines PTG from a personality perspective see Jayawickreme and Blackie [2] and associated papers; for considered discussions of both theoretical and measurement components see Park and Tennen and Affleck [5, 6]). Of relevance to the current paper, there are two main issues. First is the nature of the measurement of PTG. Briefly, owing to the elusive and random nature of the types of events that trigger PTG, it is very difficult to operationalize the construct as the actual change in positive adjustment or other psychological measures taken before the stressor and for some time afterwards and almost no researchers have done so (a key exception is the study by Frazier *et al* [7]). Park suggests that PTG may either be veridical, in that there are observable changes in factors such as world- and self-views, values and relationships, or nonverdical, in that growth is a function of illness-related cognitive processes [5]. For example, in Taylor’s cognitive adaption theory, positive illusions or temporal comparisons may represent a form of downward comparison, in which individuals maintain a high current self regard by constructing a relatively negative past self [8]. Instead, much of the field focuses on self-perceived and reported PTG (i.e., what Park would term nonveridical growth) and in turn, a substantial proportion of this research has focused on the benefit finding aspect of PTG. Participants tend to respond on standardized inventories to items that tap into beliefs such as whether since their diagnoses, they have developed new paths in life, or a closer family life [9, 10].

Second, much of the current PTG literature is cross sectional. Notwithstanding measurement issues, although several commentators (e.g., Helgeson, Jayawickreme and Tennen and Affleck) have urged researchers to conduct more longitudinal studies, comparatively few have been carried out [2, 6, 11]. Indeed, the nature of the constructs under examination strongly suggests that studies would optimally employ both long periods of chronological time and relatively more rather than fewer assessments across time. The former because most theoretical perspectives agree that time spans of years rather than months are required to allow the potential effects of both post traumatic growth and maladjustment to develop. The latter because the functional form of trajectories across time may not be a smooth linear one and so the typical default minimum of 3 or 4 assessment points that is recommended by methodologists for linear mixed models may not be sufficient.

Together, these issues have given rise to concerns about the very nature of PTG and consequently about the nature of its proposed relationships with important outcomes [12]. It is beyond the scope of the present paper to cover in depth the theoretical debate regarding the nature and the ontological status of PTG (interested readers are referred to the excellent recent papers of Sumalla and colleagues and that of Zoellner and Maerckner *[12, 13])*. Here, we examine the nature of the relationship between a key component of self-perceived PTG (i.e., benefit finding) and psychological distress. The literature on this important question is best described as contradictory and the broad pattern of results, many from cross-sectional designs, is a null relationship between PTG and psychological distress (e.g. see Zoellner [13]). In this connection, previous researchers have suggested that there are two potential models of PTG; first, PTG as an (assumed positive) outcome in its own right that, through mechanisms such as meaning making, can lead to increased adjustment and lower distress but only in time; and second, PTG as self-perceived but not actual growth which is a form of coping response to the traumatic event that could buffer the occurrence of psychological distress but could also represent a form of maladaptive avoidance coping [13].

The nature of the designs of many PTG studies has so far precluded a specific focus on the question of the nature of the relationship between PTG and psychological distress and in particular the direction of the relations across time. Studies employing longitudinal designs can help to disentangle the theoretical and measurement issues outlined here. If PTG is an outcome of a traumatic event in its own right, regardless of what mediators might be involved, we would expect to see less future psychological distress in persons who exhibited PTG compared to those who did not. By contrast, if PTG is a response to the distress of a traumatic event, we would expect to see more future self-perceived growth in those who had experienced more distress compared to less distress. In order to address such concerns, we report here on a study that contains up to 6 assessments of both psychological distress and self-perceived post-traumatic growth, measured as benefit finding, in a large cohort of colorectal cancer survivors.

We test several competing hypotheses. First, that benefit finding may be a leading indicator of psychological adjustment such that increases in benefit finding will predict improvements in psychological distress across future assessments. This hypothesis is based upon the theoretical position that PTG is a positive outcome per se that, possibly through a series of mediating mechanisms on which the present study is mute, leads to better psychosocial adjustment. Second, that psychological distress may instead be a leading indicator of benefit finding such that decreases in psychological distress will predict increasing benefit finding across future assessments. This hypothesis is based upon the theoretical position that PTG is largely illusory and may even represent a form of maladaptive, avoidance coping with respect to a stressor. The strong forms of each of these hypotheses are mutually exclusive. That is, if benefit finding predicts distress prospectively, then distress does not predict benefit finding prospectively, and vice versa. However, a weaker form is actually a third, distinct hypothesis in that psychological distress and benefit finding may each, in tandem, be a leading indicator of the other. This pattern of findings would be in keeping with two component models of PTG that contain both illusory and constructive components [13].

Accordingly, the present study used latent difference score modeling with data from a large population-based sample of colorectal cancer patients to: 1) describe the trajectory of post-traumatic growth for colorectal cancer patients from soon after diagnosis to five years subsequently 2) assess the heterogeneity of a post-traumatic growth response to cancer over time and 3) describe the simultaneous and longitudinal relationships between post-traumatic growth and psychological distress after colorectal cancer.

## Materials and Methods

### Participants and procedure

These data are from a longitudinal study of patients diagnosed with colorectal cancer, the sample and methods of which are described in detail elsewhere [14]. Ethical approval was provided by the University of Queensland. All residents in Queensland, Australia with a histologically confirmed diagnosis of a primary CRC between January 1, 2003, and December 31, 2004 were eligible. Eligibility criteria included speaking English; having no hearing, speech or cognitive disabilities that would prevent completing a telephone interview; and being aged between 20 and 80 years at diagnosis. The treating doctors of 3,626 eligible cases were approached in writing for permission to contact their patients regarding the study. Letters were re-sent to non-responding doctors two weeks after the initial mailing, and doctors were then telephoned weekly until an answer had been received.

The 3,182 cases for whom doctor consent was obtained were mailed a letter (signed by their treating doctor, explaining the study and inviting participation), a study information sheet and a consent form. Non-responders were sent a second letter two weeks later and up to two follow-up telephone calls. Age, sex, tumor site and stage of disease were collected from pathology/ medical records. Socio-demographic variables were assessed by computer-assisted telephone interview; all other measures were collected by mailed self-report survey.

In all, 1,966 participants provided informed consent and entered the study. These study participants completed a self-administered questionnaire and computer assisted telephone interview at approximately 5 months after diagnosis (Time 1 (T1)) with follow up at 12 (T2), 24 (T3), 36 (T4), 48 (T5) and 60 months (T6) post-diagnosis. Among the remaining 1,825 participants, 459 (21.2%) participants had died by five years post-diagnosis (T6). On the basis of relatively extreme survival rates (5-year survival rates of 18%) and to be consistent with other studies 40 patients with Stage IV disease were excluded from our analysis of long term outcomes [15, 16]. At T6 there were 837 participants with complete data.

The mean age of the participants was 64.63 years (Range 21 years to 81 years; SD = 10.35); 4% of participants were never married, 74% were married or in a defacto relationship, 11% were widowed and 11% divorced or separated. Most participants had between 8 and 11 years of schooling (40%) with 14% having less than 8 years, 10% had completed high school, 23% had technical college education and 13% had university education; 60% of participants were male and 40% were female.

### Outcome variables

#### Post-traumatic growth

Post-traumatic growth or benefit finding was assessed with a 16‐item scale assessing perceived positive outcomes resulting from a diagnosis of cancer [9]. Participants are asked to rate a series of statements about how their cancer has affected their lives on a five‐point scale of 1 (not at all) to 5 (extremely). The measure has three subscales: personal growth (eight items) that addresses personal strength and development; interpersonal growth (five items) that reflects increased appreciation and quality of interpersonal relationships; and acceptance of life’s imperfections (three items). Higher scores indicate a greater level of benefit finding as a result of the cancer experience. In the present study internal consistency for each subscale was excellent (personal growth α = 0.94–0.92, interpersonal growth α = 0.86–0.90, acceptance α = 0.87–0.90).

#### Psychological distress

The Brief Symptom Inventory −18 [17] was used to assess psychological distress. Respondents report the degree of distress caused by each symptom during the previous week on five‐point scales ranging from 0 (not at all) to 4 (extremely). The items of the BSI‐18 are grouped evenly into three subscales: anxiety, depression and somatisation. Internal consistency for all subscales was very good (anxiety α = 0.84–0.88; depression, α = 0.87–0.89; somatisation,α = 0.71–0.78).

### Statistical Analyses

Posttraumatic growth and psychological distress scores over the 6 longitudinal assessments were analysed with multivariate change or latent difference score models (LDS)[18]. This specialised form of structural equation model combines elements of latent growth models and autoregressive cross lagged models that are often applied in the longitudinal analysis of outcome trajectories [19]. In LDS, the observed scores on a given outcome are modelled as a latent true score plus a residual that encapsulates random error. In turn, the latent true score for an outcome is the sum of the latent true score of the same outcome, measured at the preceding assessment, and a latent difference score, representing change in the latent true score since the preceding assessment. By so estimating the latent change between assessments, change itself can be the focus of prediction. Accordingly, Eq 1 (after Grimm et al. [2012], Eq 7 [18]) shows how LDS models focus specifically on the nature of latent change as an outcome and incorporate both static and dynamic predictors of change.

$$\Delta Y{\left[t\right]}_{n}=s_{Y}+\beta_{Y}\cdot Y{\left[t-1\right]}_{n}+\gamma_{Y\cdot X}\cdot X{\left[t-1\right]}_{n}+\varphi_{Y}\cdot \Delta Y{\left[t-1\right]}_{n}+\xi_{Y\cdot X}\cdot \Delta X{\left[t-1\right]}_{n}$$ (1)

According to Eq 1, the change in the outcome at any one time of assessment (represented by the latent difference score, Δ*Y*[*t*]_*n*_) is modelled as having a fixed linear slope as a constant (i.e., expected change when all other predictors are zero; *s*_*Y*_) and, usually of more substantive interest, effects conditional on the latent true score of the same outcome at the preceding assessment (i.e., proportional change in LDS terms; *β*_*Y*_) and of a different outcome at the preceding assessment (*γ*_*Y*∙*X*_). In this way, LDS models capture the acceleration or deceleration in the trajectory of an outcome across time as a function of the true scores on the same or different outcomes at previous (i.e., lagged) time points. For example, in the present study, LDS models can address whether the expected change in psychological distress at a given assessment is either attenuated or potentiated by the level of psychological distress and benefit finding reported at the previous assessment. Eq 1 also shows that lagged *changes* (i.e., the regression parameter, *φ*_*Y*_, for the change in the outcome from *t*-2 to *t*-1, Δ*Y*[*t* − 1]_*n*_) also predict Δ*Y*[*t*]_*n*_. This introduces the concept of a leading indicator, as a variable for which change across assessments, rather than the absolute level, predicts future changes in the outcome. Again, a leading indicator can be either the outcome itself or another outcome. For example, in the present study, it may be asked whether, independent of its overall level, the amount of change in benefit finding across time (i.e., from *t*– 2 to *t*– 1), predicts future changes in psychological distress (i.e., from *t*– 1 to *t*). The concept of a leading indicator has intuitive appeal as a marker variable, the nature of whose trajectory is more important than the overall level. Finally, it is important to note that, although the examples above have used psychological distress to stand for *Y* and benefit finding to stand for *X*, LDS models with full coupling (Grimm et al, 2012) allow both processes to be modelled simultaneously as outcomes and predictors (i.e., Eq 1 is replicated with the roles of psychological distress and benefit finding reversed, respectively).

In the present study, LDS models are ideal for answering research questions about the mutual, longitudinal influence of posttraumatic growth and psychological distress in CRC survivors. As well, the analyses give rise to a series of nested models that sequentially incorporate each of the additional lagged and coupling parameters. Deviances and AIC values can be used to evaluate whether explanatory power is significantly enhanced by the addition of successive model components. For the present analyses, each step was significant for each outcome and the final steps indicated that benefit finding and psychological distress were explained best by a full model with both constant change and lagged change effects and with coupling of constant change and proportional change from the other outcome, respectively.

## Results

### Attrition analyses

An attrition analysis was conducted to compare participants who remained in the study at the final data collection point to those participants who dropped out on attributes relevant to the outcomes. The analysis was conducted twice, focusing on participants who had dropped out at the third measurement occasion and at the sixth and final measurement occasion (where 837 participants remained), respectively. However, the results and conclusions of the two analyses are extremely similar and thus only the latter is reported here. Compared to the drop outs, the participants who remained in the study at Time 6 were more likely to be female (35.65% vs 45.55%, p < .001) and less likely to have private health insurance (42.41% vs 37.15%, p = 022); and they were less likely to have been at Stage III (42.31% vs 25.04&), equally likely to have been at Stage II (35.83% vs 36.40%), and more likely to have been at Stage I (21.86% vs 38.56%, p < .001). By contrast, there were no differences in whether they were married, and neither in their educational attainment nor their ages. All analyses were conducted using maximum likelihood (in Mplus 7) to account for missingness.

### Initial analyses

The group means on the two outcomes are shown in Table 1. Means were calculated for all members of the sample who were available at each assessment and this corresponds to the sample used in the LDS models. Inspection of Table 1 shows little apparent change relative to standard deviations at the aggregated group level. However, inspection of the data and of individual participant trajectory plots suggested wide variability at the individual level. Accordingly, further analysis of the heterogeneity was undertaken with LDS models.

**Table 1:** Means and standard deviations for benefit finding and psychological distress across study timepoints.

Timepoint				95%CI	
benefit finding	N	Mean	SD	lo	hi
1	1768	3.66	0.99	3.62	3.71
2	1528	3.50	1.00	3.44	3.55
3	1273	3.54	0.97	3.49	3.60
4	1094	3.55	0.98	3.49	3.60
5	945	3.54	0.96	3.48	3.61
6	835	3.57	0.93	3.51	3.64
psychological distress					
1	1807	7.12	8.17	6.75	7.50
2	1566	6.23	7.96	5.84	6.63
3	1284	5.86	7.86	5.43	6.29
4	1040	5.70	6.93	5.28	6.12
5	942	5.21	6.88	4.77	5.66
6	817	5.37	7.50	4.85	5.88

Note. Study timepoints represent years with 1 representing study onset.

Typically, separate univariate LDS models are fit to each outcome before examining various coupling models. Here we report the initial univariate dual change model in which changes in each outcome over time are modelled by a constant change component (i.e., linear slope, *s*_*Y*_) and by recent changes in the same outcome, as the proportional change component (i.e., lagged effects, β). For benefit finding, this model fit well (CFI = .98; TLI = .99; Sample adjusted BIC = 15184.06). The model suggested that from an initial level of 3.65 (*SE* = .02) there were both a significant constant change component of 2.64 (*SE* = .26, *p* < 0.001) at each assessment but that this was in conjunction with a significant proportional change component (-.76, *SE* = .07). Thus, although underlying BF may have increased linearly at each assessment, recent increases in benefit finding predicted a relatively strong deceleration in this increase; for every 1 unit of benefit finding score at the previous time, the increase in benefit finding decreased by nearly three quarters of a unit. An analogous model for psychological distress did not fit as well (CFI = .89; TLI = .92; Sample adjusted BIC = 47423.28). Nevertheless, from an initial level of 7.14 (*SE* = .19) distress decreased constantly by 5.31 (*SE* = .42) and contained a relatively strong proportional change component of-.84 (*SE* = .06), suggesting again that recent increases in distress led to higher decreases in this outcome. Each of these models was consistent with the nature of the individual trajectories observed in the data that contained many deflections and deviations from a smooth linear pattern. Note that if certain of the present hypotheses are supported, it would imply that the univariate models are misspecified. Several outliers and potentially influential cases were identified as having extreme high scores on the GSI. These were censored and all analyses re-run. Although these evidenced no change to the results at all, analyses based on censored data are reported here.

At this point, covariate analyses were conducted to ascertain whether additional predictors needed to be controlled in later models. Relevant demographic and cancer variables were introduced as predictors of both the intercepts and the latent difference scores directly. These were: gender (represented by a dummy for female), education (using a dummy for university level), age at diagnosis, clinical stage of CRC at diagnosis (n.b., Stage IV patients had already been excluded from the sample and a dummy was constructed that distinguished stages II and III together from stage I), and a dummy representing patients with no private health insurance. These analyses showed that only gender and education were consistent predictors of the intercept and trajectory of benefit finding and that stage was a significant predictor of the intercept only. For psychological distress, only the no private health insurance dummy was a significant predictor of the intercept and trajectory. Accordingly, these characteristics were retained as covariates in later models. Although stage was a predictor only of the intercept of benefit finding, it was retained as a predictor of the latent difference scores as well, in light of its importance as a marker of the cancer survivorship experience. It is to be noted that the inclusion of the covariates made no difference to the substantive conclusions of the coupling models; although, as expected, individual parameter values changed to reflect the respecification of the underlying statistical models.

### Bivariate and full coupling models

The next set of analyses examined the combined effects of allowing first the constant change component and then the proportional change component of benefit finding to predict distress and vice versa. In effect, these analyses test whether, after having accounted for the constant change or lagged changes in a given outcome, its longitudinal trajectory is influenced by either or both of the lagged levels or changes in the other outcome, respectively. The upper panel of Table 2 shows that there were no significant effects of coupling (i.e., *γ* parameters) each outcome to the measurement of the opposite outcome at the previous timepoint. In any one year, the predicted score on psychological distress was not affected by the benefit finding score in the previous year. Similarly, the predicted benefit finding scores were not associated with the previous year’s psychological distress scores.

**Table 2:** Parameter estimates for bivariate latent difference score models.

	GSI			Benefit Finding		
*Dual Change*	parameter	95% CI		parameter	95% CI	
μ_y0_	6.43	5.87	6.98	3.52	3.41	3.62
μ_*s*_	6.76	3.37	10.15	1.98	1.46	2.50
β	-.77	-.91	-.65	-.58	-.72	-.43
γ	*-*.*71*	-1.62	.21	*-*.*01*	-.02	.01
*Full coupling*						
μ_*y0*_	6.42	5.86	6.98	3.49	3.38	3.59
μ_*s*_	5.72	1.26	9.47	1.20	.52	1.88
β	-.73	-.84	-.62	-.35	-.54	-.16
γ	*-*.*44*	-1.64	.76	*-*.*01*	-.02	.01
φ	-.15	-.29	-.01	*-*.*14*	-.43	.15
ξ	7.58	5.19	9.98	-.04	-.06	-.03

Note. Dual Change = Parameters for initial dual change models; CFA = .94, RMSEA = .064 (90%CI: .059-.069). Full coupling = parameters for final coupling model with all possible parameters. In each case, covariates were included in the model, as specified; CFA = .95, RMSEA = .061 (90%CI: .057-.066). All parameters represent unstandardized fixed effects portions of models. *μ*_*Y*0_ = fixed intercept or starting point; *μ*_*S*_ = fixed slope (i.e., constant change component); *β* = proportional change component; *γ* = coupled proportional change; *φ* = coupled constant change component; *ξ* = coupled proportional change component. Estimates in *italics* are not significant at the *p* < .05 level.

The bivariate analyses were augmented by allowing progressively more coupling between constant and lagged change components in one outcome as predictors of the opposite outcome. Each additional form of coupling provided significantly greater fit to the overall model, as evidenced by Δ-2LL values until the best fitting model was the full coupling model in which change in each outcome was predicted simultaneously lagged and constant change components for its own trajectory and by those of the opposite outcome. (The full set of analyses and fit values is available on request from the first author.) Eq 2a and 2b show the final full coupling model and the lower part of Table 2 shows the corresponding parameter estimates.

$$\Delta GSI{\left[t\right]}_{n}=s_{GSI}+\beta_{GSI}∙{GSI[t-1]}_{n}+\gamma_{GSI∙BF}∙{BF[t-1]}_{n}+\varphi_{GSI}∙\Delta GSI{\left[t-1\right]}_{n}+\xi_{GSI∙BF}∙\Delta BF{\left[t-1\right]}_{n}$$ (2a)

$$\Delta BF{\left[t\right]}_{n}=s_{BF}+\beta_{BF}∙{BF[t-1]}_{n}+\gamma_{BF∙GSI}∙{GSI[t-1]}_{n}+\varphi_{BF}∙\Delta BF{\left[t-1\right]}_{n}+\xi_{BF∙GSI}∙\Delta GSI{\left[t-1\right]}_{n}$$ (2b)

The coupling models broadly replicated the pattern observed in initial analyses for parameters common to each type of model (see Table 2). For both outcomes, the effect of lagged changes on the opposite outcome (i.e., *ξ* parameters) was significant. Thus, previous upward changes in psychological distress predicted downward changes in benefit finding. By contrast, previous upward changes in benefit finding predicted upward changes in psychological distress. As well, previous upward changes in psychological distress predicted upward changes in later psychological distress.

### Summary of trajectory effects on psychological distress and benefit finding

#### Psychological distress as an outcome

By contrast to the aggregate scores (see Table 1), the individual latent distress scores increased across the years of the study. However, the magnitude of the increase consisted of a baseline positive linear component but was also proportional to the previous year’s absolute level of distress (i.e., lagged effects of level of distress on itself), such that the higher the level of distress reported the previous year, the lower the increase in distress in the current year. This suggests that distress may have increased more steeply at first but then may have plateaued or even shown a negative slope in following years. As well, the increase in distress from year to year was contingent upon previous changes in distress itself (i.e., lagged effects of changes in distress on future distress) and upon previous changes in benefit finding (i.e., lagged effects of changes in benefit finding on distress). For any year, if benefit finding had increased from two years prior to the year before, the current year’s increase in distress was proportionally higher. In this way, benefit finding serves as a leading indicator of distress, in that increases in reported benefit finding from year to year predicted higher future increases in psychological distress. A similar pattern was observed for previous changes in distress predicting future changes in itself, except that the association was negative, showing again that upward changes in distress again downregulated future distress.

#### Benefit finding as an outcome

There are similarities between the psychological distress and the benefit finding effects. Latent benefit finding scores are also seen to increase across the years of the study; with both a baseline positive linear component but also a proportional component, representing lagged effects of absolute level of benefit finding on itself. This again suggests an underlying increase with exponential deceleration. There were no lagged effects of level of distress on changes in benefit finding. However, the lagged effects of changes in benefit finding did not predict future changes in itself. By contrast, psychological distress did serve as a leading indicator of benefit finding, such that increases in reported distress from year to year predicted lower future increases in benefit finding.

## Discussion

The results show that there is considerable heterogeneity associated with both psychological distress and benefit finding scores over time. Although we found, consistent with previous literature [2], that group means on benefit finding are relatively stable across time, the univariate model results suggest that both for benefit finding and psychological distress, the variation in individual scores contains several systematic components in the form of an underlying positive linear trend and lagged and lagged change components. However, as discussed below, our results provide clearest evidence for our third hypothesis, that benefit finding and psychological distress are mutual leading indicators of each other.

Our findings do not show a pattern that would be associated with a primarily unidirectional effect from benefit finding to psychological distress. That is, these results do not provide evidence for increased benefit finding at an earlier point of cancer survivorship as a mechanism to bring about lower psychological distress later in time. In the bivariate models, higher benefit finding scores in one year were not associated with slower acceleration of psychological distress in the following year, and this is contrary to the results of some studies in the PTG field [20]. Instead, the coupling effects suggested that the relation between benefit finding and distress was a complex one in that earlier increases in benefit finding predicted later increases in distress. Thus, participants whose self-perceived benefit finding increased earlier on tended to be those who later experienced increasing psychological distress. Similarly, psychological distress was a leading indicator of benefit finding, although the relation was an inverse one; participants whose psychological distress increased earlier on tended to be those who later experienced decreasing self-perceived benefit finding.

The finding that increases in benefit finding predicted later increases in psychological distress is consistent with the findings of Kernan and Lepore [21], who found that an ongoing search for meaning was correlated with later negative affect in their sample of women in the first 18 months after breast cancer treatment. The present findings suggest that both benefit finding and sense making, as components of PTG may have similar consequences for psychological adjustment. In an extension to the findings of Kernan and Lepore, the present results suggest that it is not only benefit finding in the early stages of cancer treatment that predicts later distress but that this longitudinal relationship is found across the six yearly assessment points in the present study [21].

The present pattern of results is also consistent with earlier work on trajectories of distress in breast cancer survivors by Helgeson and colleagues [22], in particular the study by Tomich *et al* [23], who found either that benefit finding was associated with worse psychosocial functioning after one year. By contrast, another study by Helgeson and colleagues found that benefit finding did not predict membership of any of the classes of trajectories of distress found in their sample [22]. As well, our results are consistent with recent findings concerning the relationship between PTG and posttraumatic stress by [24], who found, using a 3 wave prospective design, that higher absolute levels of post-deployment PTG were associated with more posttraumatic stress measured at 15 months after returning from military conflict in Iraq.

A possibility suggested by our results is that, consistent with the doubts expressed by Frazier and colleagues [7], changes in self-reported post-traumatic growth represent something other than changes in an underlying construct of adjustment following a traumatic event. For example, some people in our sample may have sought to enhance their sense of wellbeing specifically in regard to their CRC diagnoses, as measured by the benefit finding scale, in response to cancer–related threats to the self that would later lead to increasing trajectories of psychological distress. This explanation would be consistent with a cognitive dissonance response, in which threats to the integrity of the self have been shown to lead to a tendency to accentuate positive aspects of the threat itself [25, 26]. This interpretation would need to be tested by further research.

Study limitations include a low response rate, and as previously described non-responders differed with regard to gender, age, and disease stage, although psychological distress outcomes were similar [27]. Hence some caution is needed in generalising these results. As well, we relied on self-report of benefit finding, and it has been suggested that such measures are essentially retrospective and do not measure actual pre- to post-trauma [28]. However as the first large scale longitudinal study on benefit finding in this patient population these findings have potential to guide future research.

### Conclusion

It behoves PTG researchers to seek to more accurately establish who is experiencing post-traumatic growth. Rather than examining individual differences in self-reported post factum benefit finding, future researchers may need to focus on identifying the minority of participants who actually exhibit a rising trajectory of actual adjustment that is measured prospectively rather than by recall. The goal of these endeavours would be to establish a clear description of this group of people and of any systematic relations they share. Only then would there be a point in trying to assess the predictors of membership of this category. Such studies would benefit from continuing the use of person-centred analyses such as latent class analysis and mixture modelling [29] that may better capture the heterogeneity of underlying classes of person, rather than regression analyses, mixed or OLS, that may focus on associations between variables while ultimately aggregating across people. In this way, greater attention can be paid to uncovering the processes that may drive the longitudinal course of both psychological distress and benefit finding.
